# Making a case for endovascular approaches for neural recording and stimulation

**DOI:** 10.1088/1741-2552/acb086

**Published:** 2023-01-25

**Authors:** Brianna Thielen, Huijing Xu, Tatsuhiro Fujii, Shivani D Rangwala, Wenxuan Jiang, Michelle Lin, Alexandra Kammen, Charles Liu, Pradeep Selvan, Dong Song, William J Mack, Ellis Meng

**Affiliations:** 1 Alfred E. Mann Department of Biomedical Engineering, Viterbi School of Engineering, University of Southern California, Los Angeles, CA, United States of America; 2 Department of Neurological Surgery, Keck School of Medicine, University of Southern California, Los Angeles, CA, United States of America; 3 Neurorestoration Center, University of Southern California, Los Angeles, CA, United States of America; 4 The Lundquist Institute for Biomedical Innovation, Torrance, CA, United States of America

**Keywords:** endovascular electrode, endovascular recording, neural interface, minimally invasive

## Abstract

There are many electrode types for recording and stimulating neural tissue, most of which necessitate direct contact with the target tissue. These electrodes range from large, scalp electrodes which are used to non-invasively record averaged, low frequency electrical signals from large areas/volumes of the brain, to penetrating microelectrodes which are implanted directly into neural tissue and interface with one or a few neurons. With the exception of scalp electrodes (which provide very low-resolution recordings), each of these electrodes requires a highly invasive, open brain surgical procedure for implantation, which is accompanied by significant risk to the patient. To mitigate this risk, a minimally invasive endovascular approach can be used. Several types of endovascular electrodes have been developed to be delivered into the blood vessels in the brain via a standard catheterization procedure. In this review, the existing body of research on the development and application of endovascular electrodes is presented. The capabilities of each of these endovascular electrodes is compared to commonly used direct-contact electrodes to demonstrate the relative efficacy of the devices. Potential clinical applications of endovascular recording and stimulation and the advantages of endovascular versus direct-contact approaches are presented.

## 1. Introduction

Since the early 1970s, the field of intracranial recording and stimulation has fundamentally changed our understanding of the brain. Electrophysiological recordings with intracranial electrodes are commonly used to investigate electrical activities of neurons and functional connectivity of neural circuits [1] which are the foundation of high-level cognitive functions. Direct electrical stimulation to the cortex has been used to map the motor and sensory cortices [2]. In more recent decades, the information gained from neural recordings have spurred development of novel therapies in neurostimulation and neuromodulation for many disorders affecting the central and peripheral nervous system. The potential for improving clinical outcome is mirrored only by the breadth of ailments that are being treated including traumatic injury, movement disorders, neuropsychiatric, and neurodegenerative conditions.

In order to investigate the function and deliver therapies to the neural tissue, numerous neural electrodes have been developed to interface with the brain in a variety of ways. The most common electrodes for recording from or stimulating neural tissue are either placed in direct contact with or directly above the tissue (such as on the scalp or dura). Large, external electrodes, such as scalp electroencephalography (EEG) electrodes, are used to record electrical activity through the skin from large cortical areas, providing clinical insight into the overall health and function of different regions of the brain (see section 2.1) [3–5]. Smaller, implanted electrodes, such as electrocorticography (ECoG) and depth electrodes, can record from smaller cortical areas and subcortical areas respectively and give more localized information on the electrical activity in that region. In addition, these small electrodes can provide local stimulation to neural tissue, which is useful for the treatment of many neurologic disorders and the mapping of brain function (see sections 2.2 and 2.3) [3–14]. At an even smaller size, microelectrodes record and stimulate on a cellular level, delivering current to or recording from a small handful of cells at a time, and transmitting high resolution information to and from the tissue (see section 2.4) [3, 5, 13, 14].

Although these direct contact electrodes provide useful information for clinical and research purposes, many of them require highly invasive implantation procedures with inherent risk for complication. A minimally invasive alternative is an endovascular electrode, which is delivered to the target region via a standard catheterization procedure and does not require an open brain surgery. This type of recording is known as endovascular EEG (EV EEG) and has a comparable recording and stimulation resolution to ECoG, providing detailed information on local brain areas and with the potential to treat neurologic disorders currently treated with small, implanted electrodes [7, 9, 10, 14–20].

This paper begins with a brief review of conventional, non-endovascular devices used for neural recordings and modulation, such as scalp (EEG), epidural and subdural (ECoG), and depth electrodes (stereoelectroencephalography—SEEG, deep brain stimulation—DBS, and penetrating microelectrodes), as well as the electrophysiology associated with each technique. The remainder of this review focuses on endovascular recording and stimulation, with discussion of current research with endovascular electrodes, advances in endovascular surgery and the delivery of endovascular electrodes, and the existing and potential clinical applications of endovascular recording and stimulation devices.

## 2. Non-endovascular approaches

**Figure 1.**
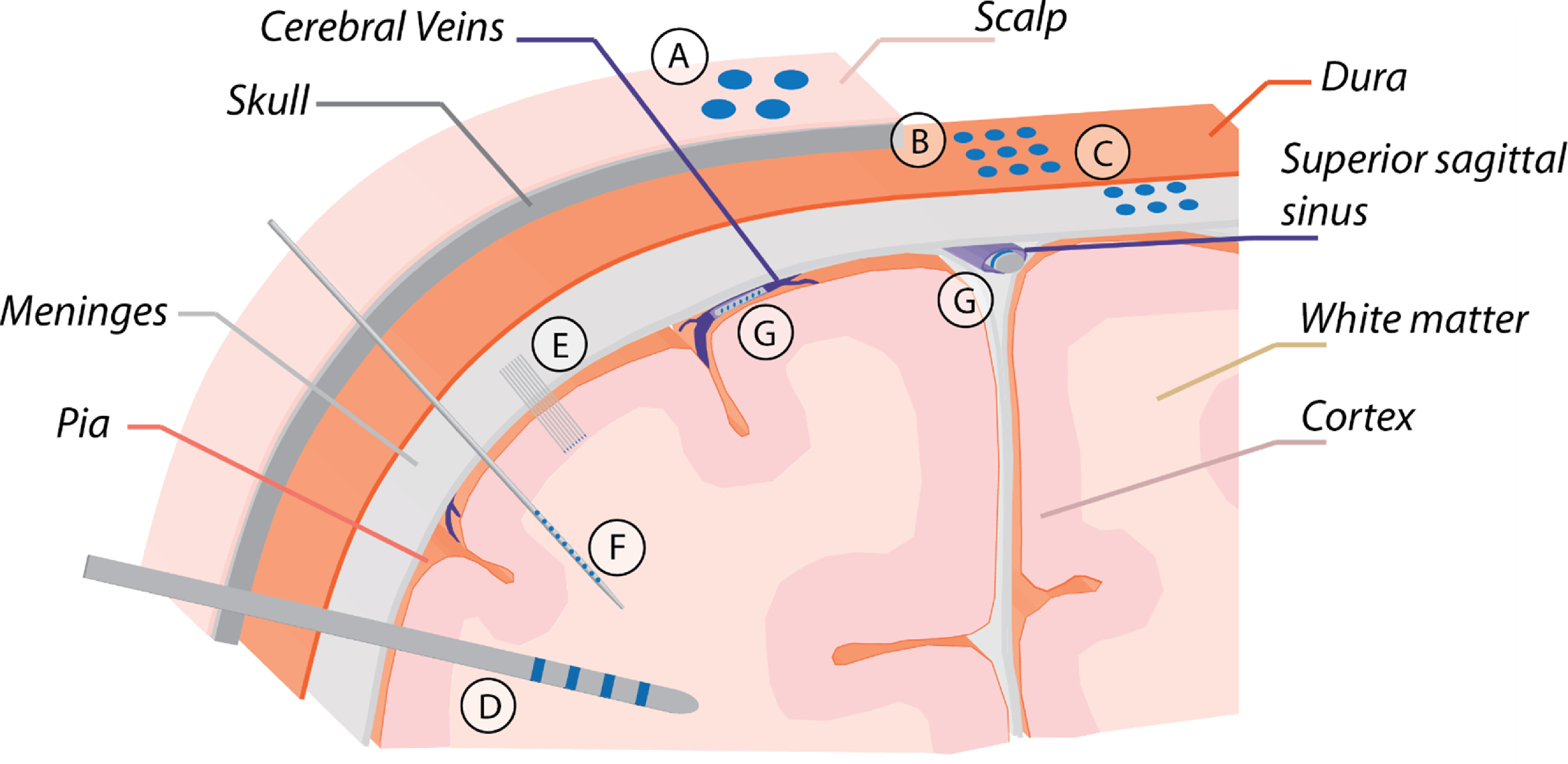
Electrode types: (A) scalp electrodes (scalp electroencephalography/EEG), (B) epidural and (C) subdural electrodes (electrocorticography/ECoG), (D) depth electrodes (stereoelectroencephalography/SEEG), (E) microwires, (F) multisite microelectrodes, and (G) endovascular electrodes.

Neurons in the brain can be accessed in many ways. There are several different types of devices used to record or stimulate neural tissue that can be categorized based on their location and placement. The type and property of signals that can be recorded with these devices depends on the electrode size and the distance and offset between the electrode and target tissue. Scalp electrodes are placed non-invasively on the surface of the scalp and record EEG signals over large brain areas. Epidural and subdural electrodes are implanted under the skull (requiring a craniotomy for placement) and are used for ECoG recording and stimulation of shallow brain areas. Epidural electrodes can also be placed on the surface of the spinal cord for spinal cord stimulation (SCS) and recording. Depth electrodes are surgically inserted into deep brain regions (DBSs) and used for SEEG and DBS. Penetrating microelectrodes (microwires, multisite probes, etc) are used for recording and stimulating small groups of neurons with single cell resolution (primarily in research settings), but also require a highly invasive procedure. Each of these electrode types is summarized in the following sections, in table 1, and in figure 1.

**Table 1.** Summary of electrode types for neural interfaces.

	Electrode type	Location	How invasive	Spatial resolution	Probe/electrode size	Sources
A	Scalp electrodes (EEG)	Surface of the scalp	Non-invasive	Large neuronal population: 1–3 cm	4–10 mm electrode diameter, multi-electrode grid	[3–5]
B	Epidural Electrodes	Surface of the dura	Invasive: Requires craniotomy	Local neuronal population: 0.5–5 mm	0.5–4 mm electrode diameter, multi-electrode grid	[3–10, 19]
C	Subdural electrodes (ECoG)	Surface of the brain	Invasive: Requires craniotomy and durotomy	Local neuronal population (large electrodes): 0.5–5 mm Local field potentials (microelectrodes): 1 mm	40 *µ*m–4 mm electrode diameter, multi-electrode grid	[3–11, 17, 19]
D	Depth electrodes (SEEG/DBS)	Deep brain tissue (penetrating)	Invasive: Requires burr hole, damages healthy tissue along probe path	Local field potentials: 1 mm	0.86–1.27 mm probe diameter, 1.3–3 mm electrode length, 4–10 electrodes	[3, 5, 9, 21]
E	Microwires	Shallow brain tissue (penetrating)	Invasive: Requires burr hole or craniotomy, damages healthy tissue along wire path	Single units: 200 *µ*m	25–80 *µ*m wire diameter, 0–20 *µ*m electrode length, single electrode	[3, 5]
F	Multisite micro-electrodes (silicon or polymer)	Shallow brain tissue (penetrating)	Invasive: Requires burr hole or craniotomy, damages healthy tissue along probe path	Single units: 200 *µ*m	10–550 × 1–50 *µ*m probe cross section, 10–55 *µ*m electrode diameter, multiple electrodes per shank	[3, 5]
G	Endovascular electrodes	Inside the blood vessels in the brain	Minimally invasive: Inserted via a catheterization procedure	Local field potentials: 1–2.4 mm	Guidewire and catheter: 0.2–0.6 mm wire diameter, 1.5–60 mm exposed wire length, single electrode	[3, 7–10, 14, 15, 17–20, 22–28]
					Microwire: 0.6–20 *µ*m wire diameter	
					Multi-electrode: 1 mm probe diameter, 1–3 mm electrode length, 40 *µ*m diameter microelectrodes	
					Stentrode: 500–750 *µ*m electrode diameter, 1.33 mm collapsed diameter	

Electrode size has the greatest impact on the frequency of signals that can be recorded. Large electrodes record averaged, low frequency electrical potentials over large brain volumes, while small electrodes record in a localized region and can detect higher frequency signals (such as local field potentials (LFPs) and unitary activities) with less spatial averaging. Signal frequency is also impacted by the amount and type of tissue between the electrode and the target. Recording amplitude is inversely proportional to the square of the distance between electrodes and target neurons, so electrodes that are placed closer to the target region can record higher amplitude (and thus higher signal to noise ratio—SNR) signals with less noise interference. As such, small electrodes must be placed very accurately, adjacent to the target in order to record a localized, high resolution signal, while large electrodes have a greater tolerance in placement accuracy but will record signals with more spatial and temporal averaging [3, 13].

Stimulation electrodes exhibit similar surface area/size and distance trends to recording electrodes: electrodes with larger surface area can deliver higher current but will stimulate a larger group of neurons, and higher stimulation amplitude is required to activate neurons with a greater distance or offset to the electrodes. In most cases, smaller electrodes with increased surface area are preferred as they can modulate localized and dedicated neuron populations which often have more clinical benefits than stimulating large brain regions.

### 2.1. Scalp electrodes (scalp EEG)

**Figure 2.**
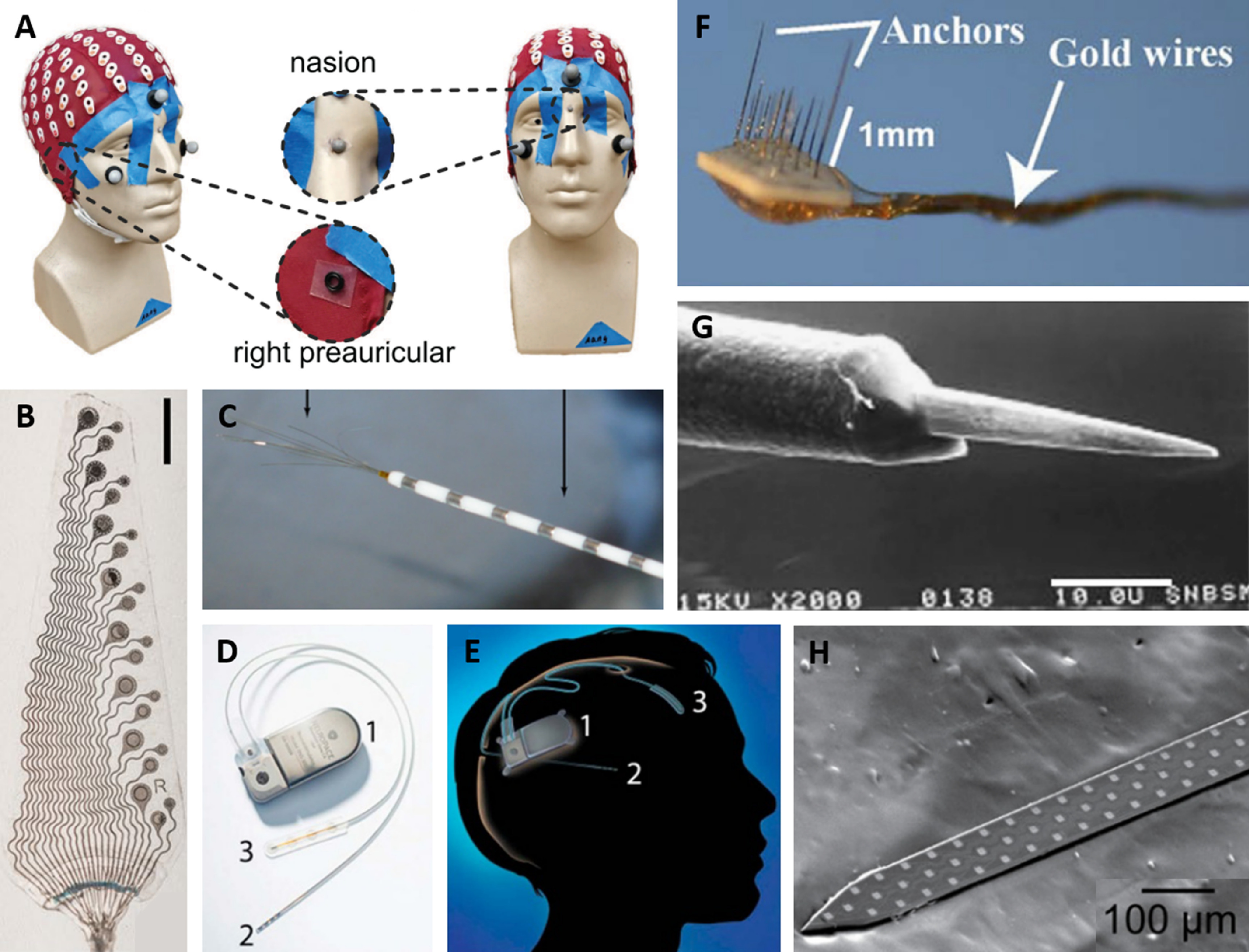
(A) Picture of a headcap with 128 scalp EEG electrodes. Reproduced from [29]. CC BY 4.0. (B) Picture of an ECoG array with 8 × 500, 750, and 1000 *µ*m electrodes. Scale bar 5 mm. Reproduced from [7]. CC BY 4.0. (C) Picture of an AdTech macro-micro electrode with outer macroelectrodes (right arrow) and center microwires (left arrow). Reproduced from [30]. © IOP Publishing Ltd. All rights reserved. (D) Picture of a Neuropace responsive neurostimulation (RNS) system, with an implanted pulse generator (1), depth probe with four ring electrodes (2), and a subdural ECoG array with four electrodes (3), and (E) a diagram showing the implanted Neuropace system. Reprinted from [31], Copyright (2015), with permission from Elsevier. (F) Picture of a microwire array with 1 mm (left) and 8 mm (right) wire lengths. (G) SEM image of a microwire electrode (exposed wire tip). Scale bar 10.0 *µ*m. Reprinted from [32], Copyright (2007), with permission from Elsevier. (H) Picture of a 64 channel silicon multisite electrode array. Reprinted from [33], Copyright (2012), with permission from Elsevier.

Scalp EEG (often referred to as EEG) is the recording of electrical signals with large electrodes (4–10 mm in diameter [5]) placed on the surface of the scalp (figures 1(A) and 2(A)). This non-invasive method is commonly used for interpretations for brain states and neurological diseases such as epilepsy.

EEG signals are classified as different bands based on frequencies for different brain activities. Most activities are associated with specific frequency ranges from 0 to 100 Hz [5]. Usually 10–128 scalp electrodes are used to cover the entire head [22, 29, 34]. Due to the thickness of the scalp and skull, electrodes are 2–3 cm away from the brain tissue, so the resulting recordings represent the average electrical activity over a large neuronal population (1–3 cm diameter region of tissue) [3, 4]. For a 129-electrode system, this results in a spatial resolution of around 6–8 cm^3^ [35]. The amplitude of a typical EEG signal is on the order of ∼100 *µ*V to several hundred microvolts.

### 2.2. Epidural and subdural electrodes (ECoG and SCS)

ECoG is the recording of electrical signals with smaller electrodes (0.5–4 mm in diameter [5–10]) placed on the surface of the dura (epidural—figures 1(B) and 2(B)) or under the dura directly on the brain surface (subdural—figures 1(C) and 2(B)). Subdural microelectrodes (∼40 *µ*m diameter [11, 17]) can also be used for higher resolution recording. Electrodes are often placed in a multi-electrode grid to cover a region of interest on the brain surface. The implantation procedure is invasive, requiring a craniotomy (and, for subdural electrodes, a durotomy) due to the size of the electrode grid, however such grids provide useful information for the localization of epileptic foci and the diagnosis of other neurologic disorders that cannot be obtained using other clinical methods.

ECoG aims to record signals with frequencies less than 300 Hz. Electrodes are much closer to the target tissue (as compared to EEG) so they are able to record a smaller neuronal population with less spatial averaging—spatial spread of subdural ECoG can be as local as around 0.5–5 mm [3, 4, 11]. The amplitude of ECoG recordings are much larger than EEG, ranging from hundreds of microvolts to several millivolts.

Subdural electrodes are also often used for stimulation of regions near the cortical surface, and epidural electrodes are used commonly in SCS. In combination with neurostimulators, these electrodes can be used for the treatment of neurologic disorders such as epilepsy, Parkinson’s and other movement disorders, or chronic pain. Subdural electrodes have also been used for closed-loop brain machine interfaces (BMIs) to provide user feedback during control of a robotic limb [31, 36, 37].

### 2.3. Depth electrodes (SEEG and DBS)

SEEG is the recording of electrical signals with small electrodes (circumferential electrodes around a probe of 0.86–1.27 mm diameter, 1.3–3 mm long [3, 5, 9, 21]) on a long probe placed into deep brain tissue (figures 1(D) and 2(D), (E)). SEEG probes contain multiple (4–10) electrodes along their length, sometimes with microelectrodes placed between larger contacts or microwires inserted into the core of the shaft to record unitary activities (figure 2(C)) [5, 30, 38]. The implantation of this device requires a burr hole on the skull with stereotactic placement of each lead. Each lead placement results in local tissue damage which in turn triggers an inflammatory response along the probe path. As is the case with ECoG, SEEG is used to localize epileptic foci or to diagnose other neurologic disorders that cannot be accurately diagnosed with other existing clinical methods.

SEEG electrodes record LFPs (signals from a small, local group of neurons) with frequencies less than 300 Hz [5, 13, 37]. Under normal conditions, the amplitude of SEEG recordings are on the order of hundreds of microvolts. The amplitude can dramatically increase to above 1 mV when a seizure occurs [39]. If placed correctly, the electrodes are very close to the target tissue, so they can record from a small, local neuronal population with less spatial averaging than EEG. Due to the small electrode size, the electrode must be within approximately 500 *µ*m of the target tissue to record LFPs, and the recorded signal is collected from an approximately 1 mm diameter area of tissue [3, 14].

Depth electrodes are also often used for stimulation of DBSs. Electrodes are connected to an implantable neurostimulator and stimulate different regions of the brain for treatment of neurologic disorders such as epilepsy, Parkinson’s and other movement disorders, or a variety of psychiatric conditions including major depressive disorder (MDD) and obsessive compulsive disorder (OCD) [31, 40].

### 2.4. Penetrating microelectrodes

To obtain neural recording at the resolution of a single cell or small group of neurons (multiple units), small diameter microelectrodes must be located in close proximity to the target tissue, as they can only record signals from up to a 200 *µ*m diameter area within approximately 50 *µ*m of the electrode surface [3, 14]. These microelectrodes can be placed on the surface of the brain (subdural microelectrodes), but more commonly penetrate the brain so they can be placed closer to the target neurons. There are several different types of penetrating microelectrodes, each with a slightly different configuration. Microwires (figures 1(E) and 2(F),(G)) are thin, insulated wires (25–80 *µ*m diameter) with an exposed tip (0–20 *µ*m tip length) that provides a single recording site. Several microwires can be arranged into a microwire array to achieve recording of unitary activities from multiple neurons within a volume of tissue. Carbon fibers have a similar configuration to microwires, with an insulated length of carbon fiber (3.5–40 *µ*m diameter) and an exposed tip (0–250 *µ*m tip length) forming a single electrode. Another common type is the silicon-based, needle-like array. For example, the Utah array is an array of insulated silicon shanks of 80 *µ*m diameter, tapered to a point with an exposed tip (50 *µ*m tip length). To achieve recording from different depths along an implanted shank, multisite electrode arrays (figures 1(F) and 2(H)) were developed. Multisite electrode arrays are planar probes (10–550 *µ*m by 1–50 *µ*m cross section) with multiple electrodes (10–55 *µ*m diameter) along the length on a silicon or polymer backbone [3, 5].

Penetrating microelectrodes require an invasive implantation procedure with a burr hole or craniotomy to access the brain tissue. Most of the time, a small part of the dura also needs to be removed. The cross-sectional area of microelectrode arrays is usually minimized to reduce damage to surrounding tissues. They are very delicate and are generally used in shallow brain areas due to insertion constraints (probes are not mechanically robust enough to be inserted into deep areas).

Penetrating microelectrodes are capable of recording single- or multi-units (signals from 1 to a few neurons) with the highest spatial resolution and cover a very broad frequency range (up to several thousands of Hz) [5, 13, 41]. The typical amplitude of spike activities recorded extracellularly using microelectrodes is from tens to hundreds of microvolts [42]. Microelectrodes have also been used for stimulation of small groups of neurons in clinical and research settings. Some examples are applications such as visual and auditory cortex stimulation to produce artificial vision and hearing, treatment of movement disorders such as Parkinson’s, and BMI [43, 44].

## 3. Endovascular approaches

While conventional approaches to neural recording and stimulation have led to significant clinical advances and research findings, an endovascular approach is preferable in many cases. Endovascular delivery of electrodes is a much less invasive procedure than the implantation of surface and depth electrodes and can provide similar recording and stimulation capabilities, even with indirect access (across the vascular wall) to the targeted tissue. Recent advances in endovascular surgical techniques and devices have made more regions of the brain accessible via endovascular access and provided safety measures to reduce risk to the patient. Novel endovascular devices are introduced into the market at an ever-increasing rate, making endovascular therapy the dominant modality for many cerebrovascular conditions. Support sheaths and distal access catheters can now provide seemingly paradoxical stiffness required for support in a co-axial system and flexibility needed to navigate tortuous vascular anatomy to reach the targeted area. Similarly, microcatheters are increasingly capable of safely reaching the distal ends of the cerebral vasculature. Novel balloon-mounted microcatheters, like the Scepter-Mini (Microvention, Aliso Viejo, CA), are miniaturizing existing balloon technology that allow for augmentation of blood flow. Micro guidewires, such as the Synchro guidewires (Stryker, Kalamazoo, MI), emphasize material softness to operate within delicate anatomy without compromising torque efficiency necessary for precise steerability.

Beyond catheters and guidewires, endovascular implants have fundamentally changed management strategies for numerous cerebrovascular diseases. Intracranial and extracranial stents are increasingly more flexible to navigate tortuous anatomy to distal targets, all the while sustaining enough radial force to keep vessels patent. Moreover, flow-diverting stents such as the Pipeline Embolization Device (Medtronic, Minneapolis, MN) and the Surpass Evolve Flow Diverter (Stryker, Kalamazoo, MI) preferentially keep blood flow within the parent vessel and away from diseased segments. Miniaturization of such flow diverting stents, such as the FRED Jr. (Microvention, Aliso Viejo, CA) allow for placements of these devices in more distal regions than ever before. Drug-eluting coatings are now being applied to stents to reduce in-stent thrombosis and distal emboli, thereby raising the safety profile of these management strategies.

These advancements in endovascular technology allow the operator to practice with increasing safety and efficacy. Combining these endovascular devices such as stents and guidewires with neural recording and stimulating techniques creates the potential for minimally invasive device delivery to areas of the central nervous system (CNS) previously challenging to reach. Subdural and epidural electrodes on the surface of the cortex fail to reach deeper cortical areas such as the sulcal folds that may represent information rich areas, and penetrating depth leads and microelectrodes cause damage to any tissue in their path, including vasculature. Endovascular delivery serves as a roadmap to accessing the cerebral venous structures that are located within these natural folds—areas previously inaccessible or at high risk for complication with existing recording techniques (see table 1 and figure 1 for a summary of types of electrodes used for neural interfaces). Researchers have examined potential vascular targets, both arterial and venous, that lie in close proximity to regions of the brain traditionally used for recording and modulatory therapy. Examples include using the middle meningeal artery for targeting lateral temporal structures, A1 division of the anterior cerebral artery for the nucleus accumbens, and cortical veins for the motor and sensory strips (tables 2 and 3).

**Table 2.** Summary of implant location and animal model for published non-specific recording studies. Blood vessel locations are illustrated in figure 4.

Location in brain	Animal	Blood vessel	Source(s)
Temporal lobe (auditory evoked potentials)	Human	Middle cerebral artery	[15, 25]
		Basilar artery	[15]
	Rabbit	Basilar artery	[23]
Parietal lobe (somatosensory evoked potentials)	Human	Middle meningeal artery	[24]
		Middle cerebral artery branches	[24]
	Baboon	Middle cerebral artery branches	[45]
	Sheep	Superior sagittal sinus	[7, 8, 10, 20, 28]
Occipital lobe (visual evoked potentials)	Rabbit	Basilar artery	[23]
	Sheep	Superior sagittal sinus	[6]
Frontal lobe	Human	Callosomarginal artery	[24]
Brain stem	Human	Basilar artery	[24]
Spinal cord	Frog	(nearby vessels)	[14, 18]

**Table 3.** Summary of implant location and animal model for published seizure mapping studies. Blood vessel locations are illustrated in figure 4.

Location in brain	Animal	Blood vessel	Source(s)
Temporal lobe	Human	Middle cerebral artery	[22, 46–48]
		Cavernous sinus	[16, 49]
		Middle meningeal artery	[50, 51]
	Baboon	Petrosal sinus	[52]
		Vein of Labbe	[52]
		Straight sinus	[52]
		Subdural sinus	[52]
Occipital lobe	Baboon	Petrosal sinus	[52]
		Vein of Labbe	[52]
		Vein of Galen	[52]
Frontal lobe	Pig	Superior sagittal sinus	[17, 19]

There are currently multiple types and configurations of endovascular recording and stimulation devices used in research settings. Because of the finite thickness of the vascular wall, endovascular electrodes generally record LFPs (<300 Hz) or other low frequency local signals (rather than single or multi units) from tissue within a few millimeters of the electrode. This type of recording has been termed EV EEG and is useful for applications such as seizure mapping and BMIs [3, 5, 13, 37].

### 3.1. Endovascular implantation techniques

Implantation techniques begin with direct access into the intravascular space, most commonly through direct needle puncture of the blood vessel wall. Factors that contribute to the selection of the arteriotomy or venotomy sites include ease of accessibility for the operator such as superficial vessels close to the skin surface, maximizing procedural safety such as selecting for compressible sites in the event of a hematoma development, and increasing patient or subject comfort including selecting vasculature appropriate in diameter to accommodate the desired introduction sheath. Once the initial vascular access has been performed, catheters can be inserted through the access sheath intro the endovascular space. The use of co-axial steerable guidewires allows for improved navigation of delivery catheters within tortuous vessel anatomy, at times against the direction of blood flow. After the delivery catheter has been advanced to the targeted vessel, the actual delivery of the device, which is often mounted onto a pusher wire, can be performed. Devices that take the form of a stent, for example, can be pushed out of the distal end of the catheter in an unsheathing technique, and in doing so, the radial force within the stent device will allow it to expand and achieve contact against the vessel intima. The intravascular space acts a natural highway for reaching deep and superficial targets with the central nervous system, all the while providing a route that can minimizes tissue injury or inflammatory response.

### 3.2. Types of endovascular recording and stimulation devices

#### 3.2.1. Guidewire/catheter electrodes: 1973–present

The earliest endovascular recording devices were based on existing guidewires and catheters. In many cases, a standard metal guidewire was advanced to the recording location and a standard catheter was used to insulate the entire length except for a few millimeters at the end, producing a single electrode at the tip [23, 53]. In some cases, custom insulated guidewires with exposed metal electrodes [15, 16, 19, 24, 46–51] or catheters with embedded electrodes [25, 52] were built specifically for recording purposes (rather than using existing guidewires, which were not insulated, or existing catheters, which did not contain electrode sites). Guidewire- and catheter-like devices used for endovascular recording typically range from 0.2 to 0.6 mm in diameter with a 1.5–60 mm exposed tip (electrode) length [15, 19, 22–25]. Although there are some publications reporting stimulation, the primary focus in most research with guidewire or catheter electrodes was recording. Only one study focused on stimulation via custom catheter electrodes [52], while all other stimulation work using guidewire or catheter electrodes focused on tissue ablation [54–56].

##### 3.2.1.1. POD catheter

In 1969, Hilal *et al* [57] developed small, magnetically guided catheters that can navigate to the intracranial vessels in man. Penn *et al* [25] modified the catheter to record electrical activity at the tip from anesthetized baboons. A 1.5 mm long and 0.6 mm diameter cobalt magnet was embedded into the tip of a catheter with a stainless steel lead down the center. A second lead was attached to a flexible spring structure that was exposed near the tip for use as a second electrode in bipolar recording.

The cobalt tip was used as an EV EEG electrode with resistance ranging from 70 to 100 Ω. Spontaneous EEG (∼50 *µ*V) was recorded while the electrode was located at the distal end of the carotid siphon from one baboon and sound-evoked EEG activity in the medial temporal lobe was recorded from another baboon with the electrode located in the middle cerebral artery. Neural signals having higher amplitude which were not present in the signal recorded with scalp electrodes (∼50 *µ*V) were observed from the recording obtained by intravascular electrodes (∼200 *µ*V). A longer EEG probe was delivered to the middle cerebral artery of a mildly sedated human patient and recorded alpha activity (mainly 14–16 Hz waves) while scalp recording showed mainly 8–9 Hz posterior alpha activity.

##### 3.2.1.2. Seeker guidewire

**Figure 3.**
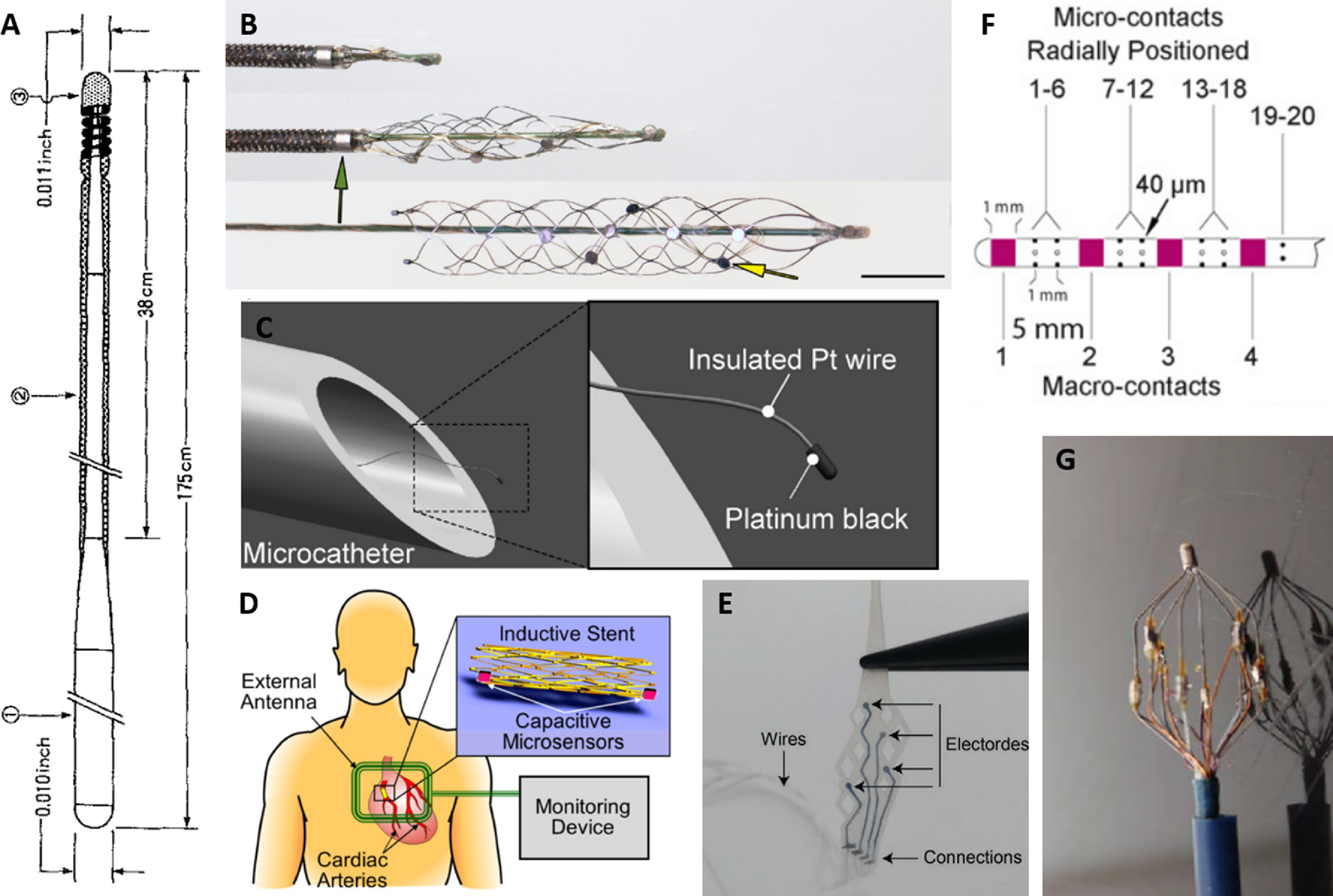
(A) Schematic of the Seeker-10 guidewire with (1) stainless steel shaft, (2) Teflon coating, and (3) a single platinum electrode at the tip. Reprinted from [15], Copyright (2012), with permission from Elsevier. (B) Picture of the Stentrode in the collapsed (top), partially-expanded (center), and expanded (bottom) configuration with platinum disc electrodes (yellow arrow) and the accompanying delivery catheter (green arrow). Scale bar 3 mm. Reproduced from [10], with permission from Springer Nature. (C) Schematic of a nanowire (0.6–1 *µ*m wire diameter) with platinum black electrode at the tip inside a microcatheter (90–300 *µ*m diameter) developed by Llinas *et al*. [14] John Wiley & Sons. Copyright © 2009 Wiley Periodicals, Inc. (D) Schematic of the antenna stent with microsensors on either end designed by Mohammadi *et al*. Reproduced from [58]. © IOP Publishing Ltd. All rights reserved. (E) Picture of the fully absorbable stent-like recording device from Fanelli *et al*. Reproduced from [59]. CC BY 4.0. © 2021 The Authors. Advanced Materials Technologies published by Wiley-VCH GmbH (F) Schematic of a multi-electrode, SEEG-like endovascular probe used by Bower *et al* with 4 × 1 mm ring electrodes and 20 × 40 *µ*m microelectrodes. Reprinted from [17], Copyright (2013), with permission from Elsevier. (G) Picture of the novel basket catheter from Gaba *et al* with 8 × 1 mm 90% platinum/10% iridium electrodes. Reproduced from [52]. CC BY 4.0.

The most commonly used guidewire in early endovascular recording studies was the Seeker guidewire—a Teflon-coated stainless steel guidewire with a single platinum electrode at the tip (275–310 *µ*m diameter, 3–60 mm length), shown in figure 3(A) [15, 24]. This device was frequently used between 1993 and 2002 for studying intracranial EEG and evoked potentials from epileptic, human patients.

Stoeter *et al* [24] used Seeker-10 guidewires (0.010″ diameter) as intra-arterial electrodes in the frontoparietal branch of the middle meningeal artery (outer surface of the temporal lobe). In 8 of 11 patients, slow alpha and theta waves were recorded with 2–4 times larger amplitude than scalp EEG electrodes (∼70 *µ*V). Nakase *et al* [15] saw a similar result when comparing intra-arterial Seeker-10 guidewire recordings in the middle cerebral artery and anterior cerebral artery (in the temporal lobe) with standard scalp EEG. In all 14 patients, a 2–5 times stronger high voltage EV EEG was recorded as compared to scalp EEG (∼100 *µ*V). In the brainstem, however, EV EEG showed no obvious differences in amplitude or frequency compared to scalp EEG.

García-Asensio *et al* [50, 51] used Seeker-10 guidewires to demonstrate that intravascular EEG recording exhibited excellent consistency (low variability between interpretations of different observers) in localizing epileptogenic areas of temporal lobe and extratemporal epilepsy with greater specificity than scalp EEG. The amplitude of intraarterial EEG was 4–5 times higher than that of the simultaneous surface EEG recording. Ishida *et al* [53] also reported more frequent spikes and sharper waves compared to scalp EEG electrodes. Boniface and Antoun [47] used a Seeker guidewire with a platinum coil at the tip (overall resistance of 33 Ω, 22 mm exposed tip) to record epileptiform midtemporal spikes and sharp waves up to 1 Hz. One third of recorded sharp waves had greatest amplitude at the intra-arterial electrode (∼300 *µ*V). Enomoto *et al* [48] also demonstrated successful recording of ictal activity in non-lesional brain areas. Kunieda *et al* [49] recorded temporal lobe activity with a Seeker guidewire in the cavernous sinus and noted similar sensitivity to ictal discharges and two times higher sensitivity to interictal discharges with EV EEG as compared to scalp EEG. Nakase *et al* [15] and Mikuni *et al* [16] used simultaneously implanted subdural strip electrodes and EV EEG electrodes in the temporal lobe to record interictal spike discharges, many of which were not detected by scalp EEG.

Seeker-10 guidewires were also used to record somatosensory and auditory evoked potentials by Stoeter *et al* [24]. Latencies of evoked peaks were similar in the intravascular and scalp recordings. Amplitude of evoked potentials showed clear dependence on the position of guidewires. Intravascular electrodes advanced to a point near the corresponding cortical regions can record evoked potentials with similar amplitude as signals recorded with conventional scalp electrodes.

##### 3.2.1.3. Other guidewire/catheter electrodes

Other commercial guidewires have been used for EV EEG in several human and animal studies. In most cases, a metal guidewire tip is used as an electrode, and the length of the guidewire (except for the tip) is insulated with either a polymer coating or with a non-conductive catheter.

As was the case with the Seeker guidewire, EV EEG recordings with other guidewires were more sensitive to electrical activity in the brain than scalp EEG. Ishida *et al* [53] and Hollanders [22] collected EV EEG recordings during the Wada test (examining language and memory) in patients with temporal lobe epilepsy or during aneurysm treatment. Endovascular electrodes regularly recorded interictal spikes that were not detected by scalp EEG electrodes and had 2–3 times higher amplitude than scalp EEG electrodes (∼5–20 *µ*V).

This trend is supported by several animal studies, including work by He *et al* [23] which showed larger amplitude, a wider frequency band, and 100 times better SNR in EV EEG versus scalp EEG in rabbit. Fujimoto *et al* [19] found that 97% of artificially generated epileptic discharges captured by subdural electrodes were also captured by endovascular electrodes in pig superior sagittal sinus (SSS).

Evoked potentials (visual, somatosensory, and auditory) were successfully recorded in baboons by Reuter *et al* [45] and Gaba *et al* [52] and in rabbits by He *et al* [23]. In rabbit, cardiac artifacts were seen in the large vessels (small vessels could not be used due to size constraints), but artifacts could be removed using simultaneous electrocardiogram recordings [23]. Gaba *et al* [52] also experimented with stimulation, noting the best results of successful capture when using balloon or basket catheters (figure 3(G)) which expanded to contact the vessel wall.

#### 3.2.2. Multi-electrode, SEEG-like probes: 1998–2013

To gain higher resolution recordings, endovascular devices with multiple electrodes along their length were developed. These devices have very similar or identical configuration to depth probes, with several ring electrodes (1–3 mm long) at the end of a catheter (∼1 mm diameter), and optional microelectrodes (∼40 *µ*m diameter) between larger contacts (figure 3(F)) [17, 26]. The center of the catheter holds the electrical leads which travel outside the body to connect to recording equipment.

Thomke *et al* [26] first introduced multi-electrode endovascular recording in 1998 with a brief report of successful EV EEG in a human subject during routine catheterization, however Bower *et al* [17] was the first to publish a detailed study using such a device in 2013. To better quantify features of intravascular recording, Bower *et al* [17] compared intravenous recording with intracranial recordings collected with a subdural electrode grid (macroelectrodes: 2 mm diameter, 5 mm separation in a 5 × 6 array, microelectrodes: 40 *µ*m diameter, 1 mm separation in 4 × 4 arrays). A standard, clinical depth electrode with four macroelectrodes (ring electrodes, 1 mm length, 5 mm separation) and 16 microelectrodes (40 *µ*m diameter, 0.5 mm separation) was used as the endovascular recording device and inserted into the SSS. Epileptiform spikes and responses to electrical stimulation of the cortical surface were recorded on intravenous electrodes in the SSS in anesthetized pigs. Epileptiform spikes recorded intravascularly had roughly the same amplitude (∼500 mV) as those obtained from subdural grids. High frequency ‘microspikes’ (duration ∼ 100 ms, amplitude ∼ 250 mV), which presumably arose from small, spatially restricted neural populations, were recorded with intravenous and subdural microelectrodes but not macroelectrodes. Such recording suggested that intravascular signals may provide sufficient spatiotemporal resolution to record a broad range of neural activities. The filtering effect of vessel walls was also evaluated by passing low-amplitude sine wave currents between macroelectrodes at opposing corners of the subdural grid. Relative frequency response curves of intravascular recordings were reduced by 11.0% at 30 Hz, 24.2% at 100 Hz, and 7.5% at 1000 Hz. Recording fidelity remained sufficient to observe epileptiform activity on the intravascular electrode.

#### 3.2.3. Micro/nanowire electrodes: 2005–2009

Microwires (thin, insulated wires with an exposed metal or conductive polymer tip) have also been utilized for intravascular recording from peripheral and central nervous systems. Microwires for intravascular use range in diameter from 0.6 to 20 *µ*m, sometimes with a larger tip diameter to produce a larger recording electrode area [14, 18, 27]. Microwires, which are relatively small compared to traditional guidewires or catheters, can be used individually in small veins or arteries (including those in small animal models) or as bundles of multiple microwires (allowing for multi-channel recording).

Llinás *et al* [18] composed an electrode system with four 20 *µ*m diameter platinum/iridium microwires and used these electrodes to record evoked activity generated by sciatic nerve stimulation within the sciatic artery in frog. Average noise level of the intravascular recording was around 20 *µ*V and the SNR of evoked activities was greater than 30. Intravascular recordings compared with signals recorded from nerve surface using 300 *µ*m silver ball electrodes showed that level and frequency characteristics of the noise were similar. The power difference was consistent with electrode size. Another electrode array comprising 2–4 insulated wires with 0.6–1 *µ*m diameter was developed and used for recording from the CNS (figure 3(C)). Each wire within the array ends in a small, bare, cup-like enlargement covered with platinum black to reduce impedance (impedance value not mentioned). *In vitro* recording of evoked activity from the spinal cord was obtained with the electrode array. Intravascular signals had higher amplitude field potentials (∼0.5–2 mV, response increased with increasing stimulation strength) and similar noise levels as surface recordings. The Llinás group later evaluated the mechanical properties of the electrode to ensure proper placement was possible in the spinal cord and confirmed the similarity of intravascular and surface recordings [14]. This group also developed a 15 × 15 *µ*m, steerable, conductive polymer strip and demonstrated that the performance of the conductive polymer electrode was comparable to silver microwires [18, 27].

#### 3.2.4. Stentrode: 2016–present

Another intravascular recording device was developed by Oxley *et al* [10] in 2016. Platinum/tungsten leads (25–33 *µ*m diameter with polyimide insulation) were welded onto the back of platinum disc electrodes (500 or 750 *µ*m diameter, 50 *µ*m thickness). Electrodes were attached to a commercial, self-expanding stent (collapsed diameter of 1.33 mm which expanded to 3–4 mm, 31.1–32 mm length) with 2–6 mm electrode pitch using a biocompatible adhesive. The leads were wrapped around a stainless steel stylet (310–410 *µ*m diameter) which was attached to the stent to aid insertion (see figure 3(B)). The full Stentrode assembly was sized to target the SSS [7–10, 20, 28, 60].

The Stentrode group performed several long term studies in sheep SSS with implantation lasting up to 190 d with the goal of producing a minimally invasive device for a BMI. Electrical performance was evaluated throughout the implantation period to determine if the Stentrode was suitable for BMI. Electrochemical impedance spectroscopy (EIS) was conducted throughout the implantation period to evaluate the electrode–tissue interface. The 1 and 10 kHz impedance doubled from bench testing immediately following implant. Low frequency impedance (<1 kHz) gradually decreased over 8 d, then remained stable for the remainder of the 13 week testing period. The 100 Hz phase angle increased for the first 7 d, then remained stable. This is likely due to the incorporation of the electrodes into the vessel wall, which increased the capacitance of the electrode-tissue interface [20, 28]. Data was not presented after 13 weeks due to insufficient remaining functional electrodes (40%–60% of electrodes failed due to lead breakage or shorting to the stent after 4 weeks; after 13 weeks there were not enough remaining functional electrodes to report statistically significant results) [8, 28]. EIS conducted post-implantation revealed that absorption and densification of surface proteins onto the electrode surface may cause low frequency (<1 kHz) phase and impedance changes of the electrode [10]. Further studies with varying electrode sizes found that low frequency (<10 kHz) impedance and phase were primarily dictated by the electrode tissue interface, while high frequency properties were dominated by electrode size [8].

Electrochemical changes during the beginning of the implantation period were primarily due to adsorption of proteins onto the electrode surface and incorporation of the electrodes into the vessel wall. This process occurred over a period of 6–30 d. Stentrodes that were implanted for a longer period of time had a larger portion of electrodes and stent struts incorporated into the lumen wall and a thicker tissue covering (strut-lumen distance). Over 190 d, the lumen area decreased slightly (due to tissue growing over the Stentrode walls), however no vessel occlusion was observed [6, 10, 20, 61].

Spatial resolution and recording stability of individual electrodes were evaluated by measuring somatosensory evoked potentials (SSEPs) and visual evoked potentials (VEPs) over time (up to 30 d). SSEPs were induced via electrical stimulation of the forelimb median nerve, and VEPs were induced via full field flash stimulation. Peak-to-peak amplitudes (∼20–55 *µ*V, duration 50–150 ms) were stable over time. Variation of waveform morphology in individual electrodes reflected recording of neural activity in discrete neuronal populations. This suggested that a spatial resolution of at least 2.4 mm was achievable with Stentrode [6, 10]. Spatial resolution of the Stentrode as compared to epidural and subdural electrodes was measured in a separate study while the animal was resting but awake. For the frequency band 8–24 Hz, subdural electrodes showed highest spatial resolution while epidural arrays had the lowest spatial resolution. No difference was observed above 24 Hz [7]. Recording sensitivity was verified by larger burst-suppression ratio in recordings at one month (0.51 ± 0.07) [6, 10]. SSEP recordings were also used to predict movement above chance level in sheep trained on a button press task (touching an illuminated button), although prediction of movement direction (left versus right) was only predicted at 58%–65%, indicating that the Stentrode may be applicable for simple BMI tasks [62].

Acute experiments were conducted to assess the power spectra and recording bandwidth of the Stentrode as compared to epidural and subdural (ECoG) electrodes. Like ECoG recording, Stentrode recording also showed a characteristic (1/*f*) frequency-dependent reduction in amplitude. Power spectra showed that Stentrode recordings and epidural recordings had similar absolute power, maximum bandwidth (∼190 Hz), and amplitudes (∼20 *µ*V), while subdural recordings had higher power, higher maximum bandwidth (∼230 Hz), and higher amplitudes at frequencies below 200 Hz [7, 10]. In sheep, the SSS is covered by dura mater, so Stentrode electrodes implanted in this location are anatomically similar to epidural electrodes [9].

Effects of electrode type, size, and location on the signal bandwidth and power were investigated. Epidural electrodes (500 and 750 *µ*m diameter), subdural electrodes (500, 750, and 1000 *µ*m diameter), and endovascular electrodes (500 and 750 *µ*m diameter) were simultaneously implanted and evaluated. Power was highest for epidural electrodes and lowest for subdural electrodes. Maximum electrode bandwidths were 216, 234, and 226 Hz for epidural, endovascular, and subdural electrodes with 500 *µ*m diameter, respectively. Two-way analysis of variance (ANOVA) showed no significant effect of either the array location or electrode size on the bandwidth. SNR of signals recorded with three types of electrodes were similar (∼1.5–2.5). However, subdural electrodes showed a wide range of values which indicate that some subdural electrodes outperformed the endovascular and epidural electrodes [7]. SNR of endovascular electrodes was found to be stable over 30 d [6]. Decoding accuracy was found to be similar between all electrode types. Overall, the performance of Stentrode was similar to an epidural array and marginally inferior to a subdural array. Chronic performance of the Stentrode was evaluated by measuring bandwidth over a period of 190 d. Maximum bandwidth was stable up to 190 d at 197.4 ± 42.0 Hz for 0–2 weeks and 196.4 ± 20.7 Hz [7, 10, 28].

One study also used the Stentrode (alongside subdural and depth electrodes) for motor cortex stimulation via the SSS and visual stimulation via the left transverse sinus. Motor responses varied between electrode types: 58% of endovascular, 66% of subdural, and 96% of depth electrodes produced a motor response. This variation is likely due to implantation location near or far from the motor cortex. Only 23% of endovascular electrodes placed behind the cruciate sulcal vein (farthest from the motor cortex) produced a response. There were no significant differences in stimulation thresholds between electrode types [9].

An early feasibility study is currently underway with a Stentrode and internal telemetry unit implanted into the SSS of two human patients with amyotrophic lateral sclerosis. Patients have been trained on mouse click actions (in combination with eye tracking for BMI cursor control) to allow text messaging, online shopping, and managing finances. Both patients were able to begin unsupervised home use 71–86 d post implant with typing/click accuracy of 93% [63].

#### 3.2.5. Other devices

In addition to traditional electrode systems, some devices with more specific functionality have been developed. One such device is the antenna stent developed by Takahata *et al* [64–66]—a stent used as an antenna to transmit sensor data from the blood stream to outside of the body (figure 3(D)). The earliest report of an antenna stent is the Stentenna in 2003, a micromachined foil that could be expanded using a balloon catheter. The Stentenna is 20 mm long, 2.8 mm wide when being deployed (flat), and 3.5 mm in diameter when expanded, with a micromachined pressure sensor attached on one end. The Stentenna was able to successfully read pressure within physiological range in a mock artery, however no *in vivo* data has been published. Mohammadi *et al* [58] later developed other helical stent designs to fix mechanical issues with the planar design that could be used for the same purpose with various sensors attached. Chow *et al* [67] tested commercial helical stents to be used as antennae for pressure reading for heart failure detection in pigs and found some power reduction while reading through tissue.

Another device that has been developed is a fully absorbable stent-like recording device (figure 3(E)). Fanelli *et al* [59] developed this device which is built on a resorbable polymeric backbone (months to years degradation time). The device has been bench tested to verify electrical properties (via EIS), deployment, and degradation time, however there is no published *in vivo* data.

### 3.3. Comparative performance of endovascular electrodes

Endovascular electrodes have been used for recording and simulation in the brain with a high degree of success. Endovascular recording electrodes show similar performance to epidural and subdural electrodes in signal amplitude, bandwidth, SNR, spatial resolution, and power [14–18], with subdural electrodes having a slightly superior performance [7, 10, 28]. When compared to scalp recordings, endovascular recordings regularly have higher amplitude (2–5×), wider frequency band, and better SNR in most regions of the brain, which leads to a greater specificity and similar sensitivity to scalp EEG when detecting temporal lobe epilepsy [15, 16, 22–25, 47, 49–51, 53]. One outlier is in the brainstem, where endovascular recordings have a similar amplitude and bandwidth to scalp EEG recordings [15]. Stimulation thresholds are also similar between endovascular, subdural, and depth electrodes when electrodes are properly placed near the target tissue [9].

## 4. Clinical application

### 4.1. Neural recording

**Figure 4.**
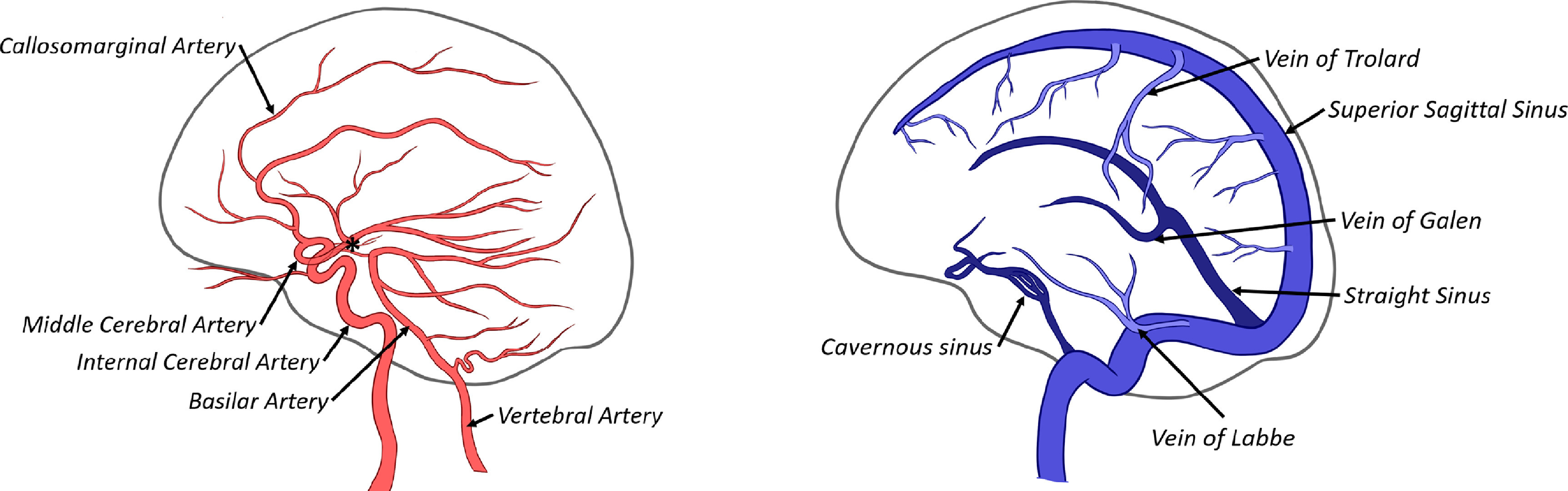
Diagrams of the cerebral arteries (left) and cerebral veins (right). Arteries and veins commonly targeted in endovascular recording and stimulation studies are labeled. The asterisk (*) denotes typical deep venous target for device placement.

Neural recording provides useful information in both research and clinical settings, either to gain insight into neural networks or to diagnose pathological conditions. Most published research on endovascular devices focuses on functionality and involves recording of background activity or evoked potentials without a specific application listed. Implant location, recording type, and animal model for each of these publications is summarized in table 2, with implant locations illustrated in figure 4. The information from these studies is useful for BMI applications, as studies overall show better recording quality from endovascular devices as compared to scalp electrodes. Several studies have directly tested the efficacy of the Stentrode for BMI and have shown promising results in sheep [62] and humans [63]. The success of these simple BMIs show promise for using the same electrodes for the control of prosthetic limbs [68].

The next largest group of published research is on the application of seizure mapping, primarily for temporal lobe epilepsy. The largest limitation of these studies is the low recording resolution—most devices used contained only a single or a few electrodes, so very limited data could be collected. Implant location and animal model for each of these studies is summarized in table 3, with implant locations illustrated in figure 4. These studies demonstrate the efficacy of endovascular recording for ictal and interictal spike detection with superior performance over scalp EEG and similar performance to ECoG.

### 4.2. Neurostimulation

Perhaps no other area of neurologic disorders has been more affected by advancements in neurostimulation than that of epilepsy. Extracranial stimulators, such as the vagal nerve stimulator (VNS), and subcortical stimulators, like DBS, present an invaluable treatment option by decreasing both frequency and severity of seizure activity in patients suffering from drug-resistant epilepsy (DRE). Open loop cortical stimulators have also been shown to reduce seizure activity when electrical stimulation is delivered to areas of the motor cortex or cerebellum. Responsive neurostimulators (RNSs), such as the Neuropace (Mountain View, CA) act as closed loop DBS where signal-detecting electric grids can be placed on the cortical surface allowing for chronic telemetry and, with enough electrical activity, penetrating depth probes can deliver predetermined stimulation to the site of epileptogenic focus. Although these conventional (non-endovascular) devices have proven to be clinically effective at treating epilepsy, endovascular devices offer the advantage of a minimally invasive and more flexible implantation procedure. Some research has evaluated the use of endovascular electrodes as neurostimulators, however more studies are required to determine their efficacy.

There is very limited data on the use of endovascular devices for neural tissue stimulation, with only a few published studies on stimulation. Gaba *et al* [52] performed stimulation of primate mesial temporal lobe and occipital lobe using four different catheters and guidewires and demonstrated successful capture of signals by recording from nearby electrodes. Opie *et al* [9] stimulated sheep motor cortex with implanted Stentrodes and reported movement in limbs, parts of the face, jaw, and neck. A cortical map was developed based on electrode locations and resulting movement response. Gerboni *et al* [6] also used a Stentrode to stimulate sheep visual cortex, verifying signal capture by recording VEPs on a simultaneously implanted subdural electrode array.

Ablation of tissue is another possible application of endovascular stimulation. A case study of accidental ablation of an epileptogenic focus as a result of a Wada test complication was published by Ammerman *et al* [56] in 2005. The ablation of the diseased tissue resolved the patient’s seizure disorder. Years later in 2008 and 2014, Henz *et al* [54, 55] published two studies on successful recording and ablation of neural tissue via the venous system in baboons and dogs.

Although DBS has not yet been attempted using endovascular electrodes, several computational studies have been performed to estimate the possibility and efficacy of performing DBS endovascularly. Endovascular DBS has potential to be a minimally invasive alternative to stereotactic DBS in the treatment of conditions such as epilepsy, Alzheimer’s disease, MDD, OCD, addiction, eating disorders, and many others [21, 69, 70]. Teplitzky [71] and Neudorfer *et al* [69] identified several common DBS targets that are located adjacent to sufficiently large blood vessels (>1–1.5 mm lumen diameter): the fornix, nucleus accumbens, subgenual cingulate white matter, ventral capsule, medial forebrain bundle, dentatorubrothalamic tract, pedunculopontine nucleus, subcallosal cingulate cortex, motor cortex, cerebellum, and thalamus. Teplitzky [71] modeled endovascular and stereotactic DBS in two of these targets (fornix and subgenual cingulate white matter) and reported comparable stimulation thresholds for the two electrode types. This result, along with similar recording characteristics of endovascular and subdural/epidural electrodes, also supports the feasibility of closed-loop DBS using endovascular electrodes [68].

Endovascular electrodes also present an alternative option for peripheral nerve stimulation. Liu *et al* [72] modeled human pelvic splanchnic nerve stimulation and found achievable stimulation thresholds depending on the distance between and relative orientation of the nerve and nearby blood vessel. Opie and O’Brien [70] reported the same result when modeling vagus nerve stimulation.

### 4.3. Common complications

During implantation of guidewire-like endovascular electrodes or the following recording period, some patients recorded complications such as retrobulbar pain or headache. In addition, there have been some reports of patients pulling out or moving a recording electrode on a guidewire during or after a seizure [16, 49]. Oxley *et al* [73] evaluated parameters associated with optimal delivery system of the Stentrode. In the ovine model, specifically, complications were almost exclusively observed when using the larger 5 or 6 French catheter system. Such complications include venous dissection and subdural hemorrhage formation. Moreover, the group noted that venous dissection tends to occur when navigating the delivery catheter around tortuous anatomy such as the confluence of sinuses. On the contrary, smaller catheters such as the 4 Fr system were able to successfully navigate the ovine venous anatomy and enter the SSS without any complications. In the two person human trial of the Stentrode, no device-related complications have been published [63].

## 5. Discussion

One of the major limitations to the existing neural recording and therapeutic modalities is the invasive nature of device implantation. Traditional access for device implantation often results in risk of direct neuronal injury or disruption of the blood–brain barrier resulting in inflammatory tissue response. Such a physiologic response may disrupt device signal detection or stimulation as well as lead hardware decomposition due to the local immune response. Opie and O’Brien [70] examined the efficacy of many of the existing neurostimulator devices such as DBS and RNS weighed against their relative medical and surgical complications in patients with DRE. This group calculated a percentage of overall benefit of a device as a function of the reported responder rate (RR), which they defined as the number of patients who saw a greater than 50% reduction in seizure frequency, while considering medical and hardware related risk. They reached a conclusion that although the RR for VNS was relatively low at 49.7%, this device was deemed the safest with a combined medical and hardware risk of 7.8%. On the contrary, direct motor cortex stimulation was seen as the most efficacious with a RR of 83.3%. Understandably, the more highly invasive nature of this device delivery, namely the need for a craniotomy and dural opening, placed the combined medical and hardware risk at a higher 12.5%. This work reflects the need for improved balance of efficacy by direct stimulation and safety by minimizing invasiveness of the therapeutic procedure. Advancements in endovascular capabilities in the treatment of a multitude of cerebrovascular disorders have created an opportunity to utilize this same technology for minimally invasive delivery of neural recoding and stimulator devices.

Endovascular electrodes have been shown to be effective for recording and stimulation in the cortex with similar resolution to subdural and depth electrodes. Several suitable large animal models have been identified (pig, baboon, and sheep, which has the most detailed review with respect to endovascular devices [73]) for testing of research devices. Small animal models have also been used, however devices can only be deployed into the larger vessels of small animals, in which cardiac artifacts are often seen [23]. In large animals and humans, cardiac artifacts are seen in the large vessels of the neck but often disappear once the electrodes are advanced into the brain [25].

Thus far, the longest published chronic implant of an endovascular device took place over 190 d (Stentrode implanted in sheep SSS [10]), other studies have taken place acutely or over the course of a few weeks. Some safety information can be drawn from bare stents (which is an established, safe procedure) and cardiac leads (which show moderate risk as the lead size increases with respect to the lumen size) [74]. Device longevity is also a concern, with Stentrode implants experiencing significant loss of active electrodes (40%–60%) within a few weeks of implant due to lead breakage or electrode shorting [8, 28], similar to early cardiac leads which experienced significant failures early on [74]. This remains the largest barrier to clinical uptake of the Stentrode (and other endovascular devices), as a high functional electrode count is necessary for proper function of the BMI system or other chronic uses.

## 6. Conclusion

Recent studies have shown that endovascular electrodes are a promising option for less invasive neural recording and neuromodulation. Endovascular devices can safely and more accurately reach depths of the brain that cannot be reached with depth electrodes. The placement of depth electrodes is subject to inherent error from patient movement between computed tomography (CT) scans or shifting of the stereotactic frame. Endovascular electrodes, however, are placed into a blood vessel with a fixed position with respect to the target area, ensuring a repeatable and reliable placement. EV EEG using a variety of different endovascular electrodes have yielded higher quality recordings than concurrent scalp EEG and similar quality to epidural and subdural ECoG (evaluated via amplitude, bandwidth, SNR, power, and spatial resolution). These recording studies cover a broad range of recording applications, primarily focusing on the recording of evoked potentials (visual, somatosensory, and auditory) for BMI and/or seizure monitoring. Although only a small body of work has studied the use of endovascular electrodes for neural stimulation, several studies have demonstrated successful stimulation of neural tissue and ablation of diseased brain tissue across the vessel wall.

These results demonstrate the potential for endovascular recording and stimulation to have numerous clinical applications, most notably as a treatment or diagnostic tool to replace the use of depth (SEEG or DBS) probes or subdural/epidural (ECoG) electrodes. Currently, SEEG, DBS, and ECoG electrodes are used for the treatment of a multitude of neurologic disorders, however the implantation of these devices is accompanied by significant risk to the patient. The use of endovascular electrodes will likely significantly decrease the risk to the patient and yield a similar clinical result when the target tissue is near a sufficiently large blood vessel.

Although endovascular electrodes have been shown to be a promising option in the current body of work, more research is necessary to improve existing endovascular devices, determine the longevity of devices, and assess the long-term safety during chronic implantation prior to use in clinical applications. Smaller endovascular devices with a higher electrode count (for high resolution recording and stimulation) must be developed to safely reach smaller blood vessels in the brain while preventing vessel occlusion or injury. Existing work has also primarily focused on recording, with very few published stimulation studies. While stimulation has been demonstrated with guidewires and the Stentrode, the safety and efficacy of stimulation must be further evaluated. Stimulation-focused devices may also benefit from different electrode materials (such as platinum/iridium alloy), nanostructuring, or coatings (such as conductive polymers), which have been widely used in conventional neural electrodes [75, 76], to allow for stronger stimulation strength.

In addition, long term implant studies must be performed to assess the safety of chronically implanted endovascular electrodes and to ensure device longevity while implanted. Chronically implanted devices must be securely tethered in place and must include an implanted telemetry unit or antenna for data and power transfer. Although the challenges of chronic devices are currently being evaluated in the early feasibility study of the Stentrode [63], more work is needed to evaluate the safety and lifetime of chronically implanted wires and electronics in different configurations and implant locations.

## Data Availability

No new data were created or analyzed in this study.
